# Relationship of the Endothelial Activation and Stress Index with 28-day mortality in urosepsis patients: a retrospective two-cohort investigation

**DOI:** 10.3389/fmed.2026.1761104

**Published:** 2026-05-12

**Authors:** Jiaqi Tang, Hu Li, Zhuo Zhang, Jiaxin Cao, Hu Nie

**Affiliations:** Emergency Department, West China Hospital of Sichuan University, Chengdu, China

**Keywords:** adverse outcomes, biomarker, Endothelial Activation and Stress Index, risk stratification, urosepsis

## Abstract

**Background:**

The Endothelial Activation and Stress Index (EASIX) is a novel biomarker for assessing endothelial dysfunction. This study aimed to evaluate its prognostic value for 28-day mortality in patients with urosepsis.

**Materials and methods:**

We conducted a retrospective study of patients with urosepsis admitted to the ICU using data from the MIMIC-IV database and West China Hospital. Restricted cubic spline (RCS) regression, multivariable Cox regression, and Kaplan–Meier analysis were used to assess the association between EASIX and short-term mortality. Four machine learning feature selection methods (LASSO-COX, Boruta, random forest, and gradient boosting) were applied to identify key prognostic features and develop a predictive model, which was evaluated using ROC curve analysis and validated in an external cohort.

**Result:**

A total of 2,593 patients were included. The 28-day ICU and in-hospital mortality rates were 16.0 and 17.2%, respectively. Higher EASIX scores were significantly associated with increased mortality across quartiles (ICU mortality: 10.5% in Q1 to 30.4% in Q4, *p* < 0.001). In the fully adjusted model, each unit increase in EASIX was associated with a 7% higher risk of ICU mortality (HR 1.07, 95% CI 1.05–1.11, *p* < 0.001), and patients in Q4 had a 57% higher risk than those in Q1 (HR 1.57, 95% CI 1.09–2.26, *p* = 0.016). RCS analysis revealed a non-linear relationship between EASIX and mortality. The predictive model incorporating EASIX, Charlson Comorbidity Index, RDW, and SAPS II achieved AUC values of 0.70–0.73 across training, internal validation, and external validation cohorts, demonstrating improved performance compared to traditional severity scores.

**Conclusion:**

EASIX is independently associated with short-term mortality in patients with urosepsis and may serve as a valuable tool for risk stratification following further validation.

## Introduction

Sepsis, a complex systemic inflammatory response to infection, remains a leading cause of mortality in non-cardiac intensive care units (ICUs) (1, 2). This syndrome can originate from various sites, with genitourinary tract infections accounting for approximately 9 to 31% of cases. Sepsis arising from such infections is specifically termed urosepsis. As a severe complication of urinary tract infections (UTIs), urosepsis poses a significant threat to patient health and imposes a substantial burden on public health systems (3, 4). In recent years, the increasing incidence of urological diseases and related surgical procedures, coupled with the growing challenge of antimicrobial resistance, has further exacerbated the burden of urosepsis (3, 4). Therefore, investigating the risk factors for UTI-related bloodstream infections and identifying reliable biomarkers for the early detection of high-risk patients are crucial for enhancing preventive strategies and improving clinical outcomes.

The Endothelial Activation and Stress Index (EASIX) is an emerging biomarker that quantifies endothelial cell activation and damage in a non-invasive and straightforward manner. The formula, originally proposed by Luft et al. (5), combines these three parameters multiplicatively as EASIX = [LDH (U/L) × Creatinine (mg/dL)] / Platelet count (×10⁹/L). It is calculated from three readily available laboratory parameters: lactate dehydrogenase (LDH), serum creatinine, and platelet count (PLT), each reflecting a distinct aspect of endothelial function. Elevated LDH, a marker of cellular injury, indicates the lysis of endothelial cells under inflammatory conditions. Serum creatinine levels reflect renal function, where impairment often signals endothelial injury leading to renal microvascular ischemia or thrombotic microangiopathy (TMA) (6–9). A decreased PLT count signifies consumptive loss due to endothelial activation and the subsequent upregulation of procoagulant factors (10). While these parameters do not directly measure endothelial injury, they reflect downstream consequences of endothelial dysfunction—including cellular injury (LDH), end-organ renal impairment (creatinine), and consumptive coagulopathy (platelets)—and collectively provide an integrated assessment of the severity of sepsis-associated endothelial stress. Endothelial cells are central to the pathophysiology of sepsis. Upon pathogen invasion, activated endothelial cells increase vascular permeability by modulating intercellular junctions. This allows adherent immune cells to transmigrate into the tissue interstitium and reach the site of infection to clear pathogens (11–14). A growing body of evidence links endothelial function, the inflammatory burst, and the oxidative stress storm to the morbidity and mortality of urosepsis, underscoring the importance of early risk assessment and intervention upon ICU admission (15, 16). Therefore, translating this complex pathophysiology into an easily calculable clinical tool, such as EASIX, may help identify patients who have already developed substantial endothelial injury and are therefore at higher risk of adverse outcomes.

Originally validated in hematological settings, particularly among patients undergoing allogeneic hematopoietic stem cell transplantation (allo-HSCT) (16), the application of the Endothelial Activation and Stress Index (EASIX) has since expanded to various critical illnesses, including cardiovascular and cerebrovascular diseases and severe asthma, demonstrating its unique prognostic value (17, 18). Nevertheless, the prognostic significance of this biomarker, which integrates the full spectrum of endothelial injury—from cellular damage and organ dysfunction to coagulopathy—remains unclear in patients with urosepsis. To address this knowledge gap, this study leverages the MIMIC-IV database and a real-world clinical cohort to systematically evaluate the association between EASIX and short-term outcomes in these patients through a retrospective study. Our aim is to provide an evidence base for improving risk stratification and personalized management in this population.

## Materials and methods

### Data source

This retrospective, two-cohort study derived its internal training cohort from the Medical Information Mart for Intensive Care (MIMIC-IV, v3.1) database. This large, open-source clinical database, maintained by the Massachusetts Institute of Technology (MIT), contains comprehensive, high-quality medical information from patients admitted to intensive care units (ICUs) at Beth Israel Deaconess Medical Center (BIDMC) (19). The extracted variables included baseline demographics, overall health status, imaging and laboratory results, comorbidities, medication history, and diagnostic information. The use of this de-identified retrospective data was granted a waiver of informed consent by the Institutional Review Board (IRB). The external validation cohort consisted of patients with urosepsis admitted to the Emergency Intensive Care Unit (EICU) of West China Hospital of Sichuan University between April 2022 and April 2024. This part of the study was approved by the Biomedical Ethics Committee of West China Hospital.

### Study population

The study population comprised adults admitted to the ICU with sepsis secondary to a urinary tract infection (UTI). Urosepsis was defined according to the Sepsis-3 criteria, which require both: (a) the presence of sepsis, operationalized as an increase in the Sequential Organ Failure Assessment (SOFA) score of ≥ 2 points from baseline; and (b) confirmation of a urinary tract infection as the source of sepsis. In the internal MIMIC-IV cohort, UTIs were identified using ICD-9 and ICD-10 diagnosis codes. In the external validation cohort (West China Hospital), UTIs were confirmed based on clinical signs and symptoms consistent with UTI (e.g., fever, flank pain, dysuria) and/or positive microbiological cultures from urine or blood specimens. The inclusion criteria were: (1) age ≥ 18 years; (2) an ICU stay and overall survival both exceeding 24 h; (3) available measurements of lactate dehydrogenase (LDH), serum creatinine (CRE), and platelet (PLT) count; (4) first-time admission to the ICU during the study period; and (5) a Sequential Organ Failure Assessment (SOFA) score ≥ 2 at admission.

### Variable extraction

Data extraction was performed using PostgreSQL (v13.7.2), Navicat Premium (v16.0), and Structured Query Language (SQL). The extracted variables were categorized into six groups: (1) demographics, (2) comorbidities, (3) vital signs, (4) laboratory parameters, (5) disease severity scores, and (6) treatment regimens. A detailed list of variables is provided in a >Supplementary Table S1. To minimize bias, variables with a missing data rate exceeding 30% were excluded from the analysis. For variables with less than 30% missing data, we employed multiple imputation using the “mice” package in R.

### Definition of EASIX and endpoints

The EASIX score was calculated using the following formula (8):


$$\begin{array}{l} \text{EASIX}=[\begin{array}{l} \text{Lactate Dehydrogenase}\;\\ (\mathrm{LDH},U/L)\times \text{Creatinine}\;\\ \left(\mathrm{CRE},\mathrm{mg}/\mathrm{dL}\right) \end{array}]/\text{Platelet}\;\\ \text{count}\;(\mathrm{PLT},\times 10n/L) \end{array}$$

The primary endpoint was 28-day ICU mortality, and the secondary endpoint was 28-day in-hospital all-cause mortality.

### Subgroup analysis

Subgroup analyses were performed according to pre-specified criteria, including age (>65 years vs. ≤65 years), sex, ethnicity, and the following comorbidities: hypertension, acute kidney injury (AKI), chronic kidney disease (CKD), diabetes, hyperlipidaemia (HLD), myocardial infarction (MI), ischaemic heart disease (IHD), and chronic obstructive pulmonary disease (COPD). Within each subgroup, a Cox proportional hazards regression model was applied for assessment, and forest plots were used to visually present the hazard ratios (HRs) along with their 95% confidence intervals (CIs).

### Feature variable selection and risk prediction model development and validation

The internal cohort was randomly divided into training and validation sets at a ratio of 7:3. In the training set, we applied four machine learning feature selection methods—Boruta algorithm, LASSO regression, Random Forest, and Gradient Boosting—to identify features associated with 28-day ICU mortality. These methods were selected for their complementary strengths: Boruta identifies all relevant features via shadow variable comparison; LASSO performs regularization and variable shrinkage; Random Forest provides importance rankings based on impurity reduction; and Gradient Boosting captures complex non-linear interactions. By intersecting the features identified by all four methods, we aimed to obtain a robust set of core prognostic variables stable across different methodological assumptions. Specifically, hyperparameters for Boruta were set as confidence level = 0.01, maximum runs = 100, with Bonferroni correction applied; LASSO regression used 10-fold cross-validation to select the optimal regularization parameter *λ*, with lambda.min as the criterion; Random Forest parameters were set as random seed = 1, number of trees = 100, max depth = 3, minimum samples per split = 2, minimum samples per leaf = 1; Gradient Boosting parameters were set as random seed = 1, loss function = “squared,” learning rate = 0.1, boosting iterations = 100, split criterion = “friedman_mse,” minimum samples per split = 2, minimum samples per leaf = 1. These hyperparameters were selected based on a combination of grid search optimization and established defaults to balance model performance with interpretability and avoid overfitting. Variables identified by all four methods were considered core features significantly associated with patient prognosis. Subsequently, multivariable Cox regression was used to identify independent predictors and construct a risk prediction model. The risk score was calculated using the following formula:


$$\begin{array}{l} \text{Risk Score}=(\text{Variable}₁\times \beta ₁)+(\text{Variable}₂\times \beta ₂)\\ +\ldots +(\text{Variablen}\times \mathrm{\beta n}) \end{array}$$

Where, β represents the regression coefficient. The predictive performance of the model was evaluated using receiver operating characteristic (ROC) curves and the area under the curve (AUC). Furthermore, the robustness of the model was validated in an external cohort with distinct patient characteristics.

### Statistical analysis

Continuous variables are presented as median with interquartile range (IQR). Group comparisons were performed using the Student’s t-test, ANOVA, Mann–Whitney U test, or Kruskal-Wallis test, as appropriate based on data distribution. Categorical variables are expressed as frequencies and percentages, and compared using the Pearson Chi-square or Fisher’s exact test. The association between variables and 28-day mortality was first assessed using univariable analysis. Subsequently, multivariable Cox proportional hazards regression was employed to evaluate the independent prognostic value of the EASIX score, with results reported as hazard ratios (HRs) and 95% confidence intervals (CIs). The multivariable analysis involved three sequential models: Model 1 was unadjusted; Model 2 was adjusted for age, sex, ethnicity, and comorbidities (including hypertension, AKI, CKD, and HLD); Model 3 was further adjusted for a comprehensive set of variables selected based on clinical relevance and univariable analysis results (see >Supplementary Tables S1–S3). Specifically, Model 3 included severity scores (SOFA, APS III, SAPS II, OASIS, Charlson, APACHE II), hemodynamic parameters (NBPS, NBPD), laboratory parameters (RDW, WBC, ALB, AG, K, TCO₂, Lac, pH, PO₂, APTT, TB, URE, LDH), and therapeutic interventions (Sa, Vp, and CRRT). To avoid multicollinearity, we calculated variance inflation factors (VIFs) for all variables in the final model, and variables with VIF > 5 were excluded (see >Supplementary Table S4). After full adjustment for potential confounders, the potential nonlinear relationship between the EASIX score and 28-day mortality was explored using restricted cubic splines (RCS) with knots placed at the 5th, 35th, 65th, and 95th percentiles. All analyses were conducted in the R statistical environment (version 4.2.2). A two-sided *p*-value < 0.05 was considered statistically significant.

## Result

### Baseline characteristics of study participants

**Figure 1:**
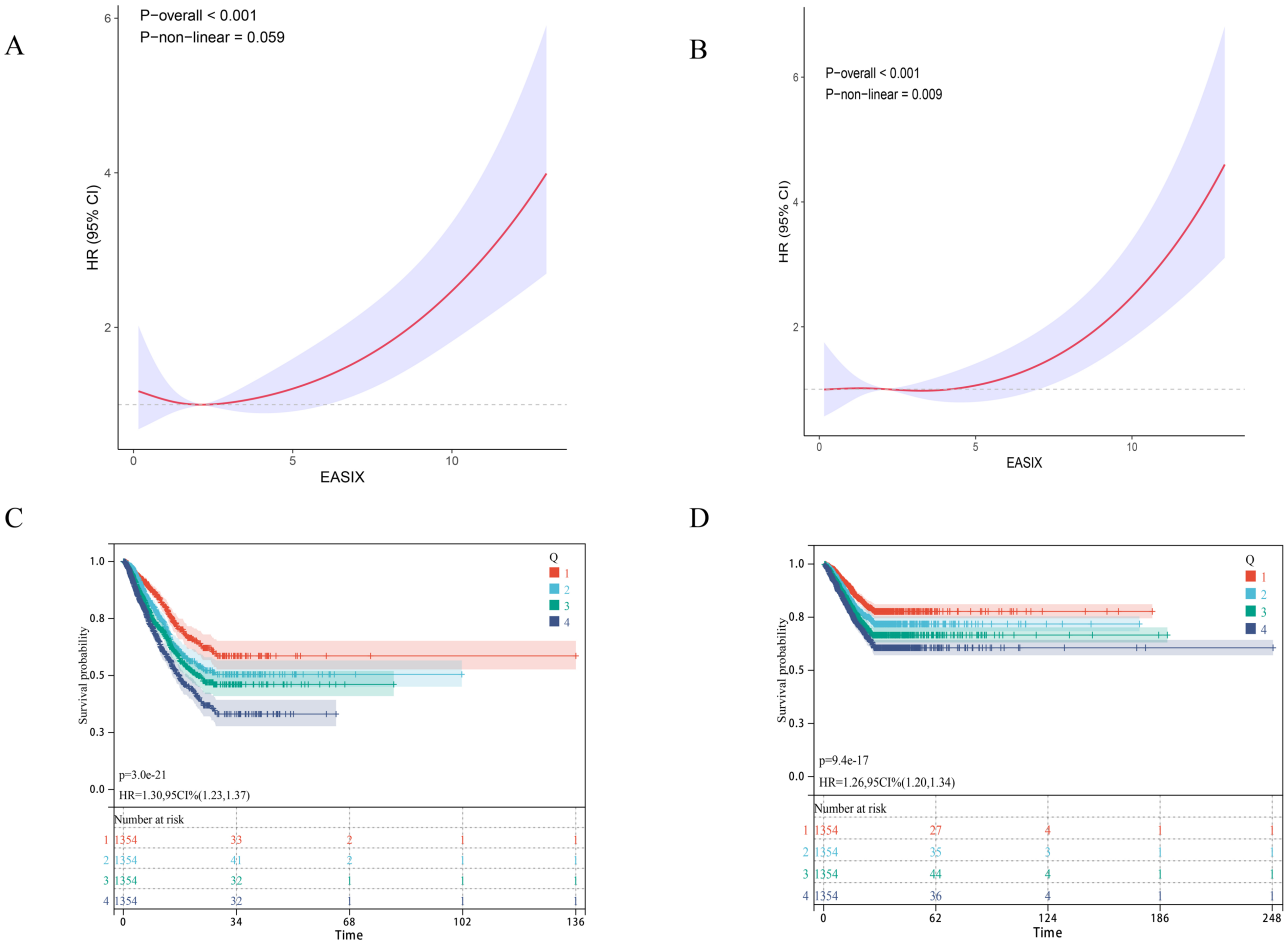
Association between EASIX and 28-day survival in the internal cohort. **(A,B)** Restricted cubic spline (RCS) curves show the nonlinear relationship between continuous EASIX score and the odds of (A) 28-day ICU mortality and **(B)** 28-day in-hospital mortality. The solid line represents the adjusted odds ratio (OR), with the dashed lines indicating the 95% confidence interval. **(C,D)** Kaplan–Meier survival curves illustrate the probability of survival from **(C)** ICU death and **(D)** in-hospital death 28 days, stratified by EASIX score stratification (Q1, blue; Q2, green; Q3, dark green; Q4, red). *p*-value and Hazard ratios (HR) with 95% confidence intervals are derived from Cox proportional hazards models, using the Low EASIX group as reference.

Based on the inclusion and exclusion criteria, 2,593 patients with urosepsis from the MIMIC-IV database were included in the final analysis. The 28-day ICU and in-hospital mortality rates were 17.2 and 16.0%, respectively. Table 1 details the baseline characteristics of the patients, categorized by EASIX quartiles. Patients were stratified into four subgroups (Q1–Q4) based on EASIX quartiles, from lowest to highest. The analysis revealed a clear gradient of increasing disease severity with higher EASIX scores. From Q1 to Q4, patients were older, included a higher proportion of males, and had a progressively greater prevalence of comorbidities such as hypertension, acute kidney injury, chronic kidney disease, and ischemic heart disease (all *p* < 0.001). Disease severity scores, including SOFA, APS III, Charlson Comorbidity Index, and APACHE II, increased stepwise across the quartiles, indicating more severe organ dysfunction and worse baseline health in the Q4 group. Laboratory findings in the Q4 group indicated pronounced inflammation and inadequate tissue perfusion: hemoglobin and platelet counts were significantly lower, while markers of renal function and coagulation (lactate, creatinine, blood urea nitrogen, and INR) were markedly elevated (all *p* < 0.001). Of note, a progressive worsening of renal function was observed across EASIX quartiles, with median creatinine increasing from 0.85 mg/dL in Q1 to 2.75 mg/dL in Q4, and median urea nitrogen from 21.5 mg/dL to 53.8 mg/dL (all *p* < 0.001), indicating that patients in higher EASIX quartiles had more advanced underlying renal impairment. Regarding treatment interventions, the Q4 group required continuous renal replacement therapy at a much higher rate than the other groups. Clinical outcomes also differed significantly, with both 28-day in-hospital and ICU mortality rates in the Q4 group being substantially higher than in the lower quartiles (all *p* < 0.001). Clinically, these findings demonstrate that patients in the highest EASIX quartile (Q4) represent a distinct high-risk population characterized by: (1) greater comorbidity burden, particularly renal impairment; (2) more severe organ dysfunction as reflected by higher severity scores; (3) more pronounced laboratory abnormalities consistent with tissue hypoperfusion and coagulopathy; (4) higher treatment intensity (e.g., CRRT); and (5) approximately threefold higher short-term mortality compared to patients in the lowest quartile. This gradient across EASIX quartiles supports the potential utility of EASIX as a simple tool for early risk stratification at ICU admission. Furthermore, trend analysis confirmed a dose–response relationship between EASIX and both 28-day ICU and in-hospital mortality (*p* for trend < 0.05; Figure 1). Restricted cubic spline (RCS) analysis demonstrated a non-linear relationship between EASIX and short-term mortality in urosepsis patients (non-linear *p* < 0.001 for both ICU and in-hospital mortality; Figures 1A,B). Similarly, Kaplan–Meier survival curves confirmed a significant positive correlation between higher EASIX scores and increased mortality. Compared to the low EASIX group (Q1), the high EASIX group (Q4) had significantly higher risks of 28-day ICU mortality (HR = 1.30, 95% CI: 1.23–1.37) and 28-day in-hospital all-cause mortality (HR = 1.26, 95% CI: 1.20–1.34; Figures 1C,D).

**Table 1:** Summary descriptives table by groups of EASIX group.

Variable	[ALL]	Q1	Q2	Q3	Q4	*p*-value
	*N* = 2,593	*N* = 648	*N* = 648	*N* = 648	*N* = 649	
EASIX	3.21 (2.92)	0.67 (0.25)	1.56 (0.32)	3.10 (0.65)	7.48 (2.47)	<0.001
Age	70.9 (15.0)	69.3 (15.5)	72.4 (14.9)	71.9 (14.1)	69.9 (15.2)	<0.001
Gender						<0.001
F	1,476 (56.9%)	439 (67.7%)	352 (54.3%)	339 (52.3%)	346 (53.3%)	
M	1,117 (43.1%)	209 (32.3%)	296 (45.7%)	309 (47.7%)	303 (46.7%)	
Race						0.015
Other races	880 (33.9%)	203 (31.3%)	247 (38.1%)	200 (30.9%)	230 (35.4%)	
WHITE	1713 (66.1%)	445 (68.7%)	401 (61.9%)	448 (69.1%)	419 (64.6%)	
Weight	82.5 (28.1)	78.6 (32.4)	81.7 (25.4)	84.4 (26.6)	85.3 (26.8)	<0.001
Hypertension						<0.001
No	1,689 (65.1%)	345 (53.2%)	405 (62.5%)	445 (68.7%)	494 (76.1%)	
Yes	904 (34.9%)	303 (46.8%)	243 (37.5%)	203 (31.3%)	155 (23.9%)	
AKI						<0.001
No	981 (37.8%)	420 (64.8%)	263 (40.6%)	172 (26.5%)	126 (19.4%)	
Yes	1,612 (62.2%)	228 (35.2%)	385 (59.4%)	476 (73.5%)	523 (80.6%)	
CKD						<0.001
No	1817 (70.1%)	573 (88.4%)	469 (72.4%)	392 (60.5%)	383 (59.0%)	
Yes	776 (29.9%)	75 (11.6%)	179 (27.6%)	256 (39.5%)	266 (41.0%)	
Diabetes						0.019
No	1,620 (62.5%)	434 (67.0%)	403 (62.2%)	403 (62.2%)	380 (58.6%)	
Yes	973 (37.5%)	214 (33.0%)	245 (37.8%)	245 (37.8%)	269 (41.4%)	
HLD						0.007
No	1,659 (64.0%)	440 (67.9%)	382 (59.0%)	412 (63.6%)	425 (65.5%)	
Yes	934 (36.0%)	208 (32.1%)	266 (41.0%)	236 (36.4%)	224 (34.5%)	
IHD						<0.001
No	1,580 (60.9%)	460 (71.0%)	404 (62.3%)	358 (55.2%)	358 (55.2%)	
Yes	1,013 (39.1%)	188 (29.0%)	244 (37.7%)	290 (44.8%)	291 (44.8%)	
COPD						0.174
No	2,139 (82.5%)	528 (81.5%)	524 (80.9%)	534 (82.4%)	553 (85.2%)	
Yes	454 (17.5%)	120 (18.5%)	124 (19.1%)	114 (17.6%)	96 (14.8%)	
SOFA	6.62 (3.22)	5.15 (2.68)	5.72 (2.70)	7.04 (2.98)	8.57 (3.35)	<0.001
APSIII	57.8 (20.2)	52.1 (18.9)	54.0 (19.5)	59.1 (18.5)	65.9 (21.0)	<0.001
SAPSII	44.5 (13.2)	40.8 (12.6)	42.5 (12.6)	45.7 (12.6)	48.9 (13.6)	<0.001
OASIS	35.7 (8.25)	36.1 (7.83)	35.2 (7.84)	35.8 (8.30)	35.8 (8.97)	0.216
Charlson	6.37 (2.87)	5.43 (2.67)	6.21 (2.89)	6.77 (2.85)	7.05 (2.81)	<0.001
APACHEII	21.6 (6.72)	19.4 (6.09)	20.1 (6.47)	22.4 (6.41)	24.4 (6.71)	<0.001
HR	91.8 (21.3)	93.6 (21.6)	91.0 (21.7)	90.3 (20.3)	92.3 (21.2)	0.026
NBPS	120 (25.5)	122 (26.8)	121 (24.9)	119 (24.7)	117 (25.3)	0.005
NBPD	67.8 (20.1)	69.6 (21.1)	67.5 (19.6)	67.1 (18.8)	67.1 (21.0)	0.088
RR	20.2 (6.45)	20.4 (6.13)	20.3 (6.62)	20.1 (6.41)	19.9 (6.63)	0.495
SpO_2_	96.5 (4.75)	96.4 (5.17)	96.7 (4.29)	96.4 (4.50)	96.3 (4.97)	0.377
Temperaturef	98.0 (4.27)	98.0 (4.45)	98.0 (4.41)	98.0 (4.12)	97.9 (4.09)	0.943
HCT	31.3 (6.61)	31.8 (6.13)	32.2 (6.52)	31.2 (6.71)	29.9 (6.87)	<0.001
HB	10.1 (2.19)	10.3 (2.06)	10.4 (2.20)	10.0 (2.18)	9.68 (2.25)	<0.001
PLT	210 (117)	294 (133)	218 (102)	182 (85.4)	144 (83.2)	<0.001
RDW	16.1 (2.54)	15.6 (2.41)	15.7 (2.34)	16.1 (2.46)	16.8 (2.76)	<0.001
RBC	3.40 (0.78)	3.52 (0.75)	3.52 (0.75)	3.38 (0.78)	3.20 (0.79)	<0.001
WBC	13.9 (15.1)	13.8 (7.41)	14.3 (20.9)	13.1 (11.3)	14.4 (17.1)	0.326
ALB	2.90 (0.62)	2.89 (0.65)	2.89 (0.63)	2.89 (0.59)	2.92 (0.64)	0.894
AG	15.3 (4.55)	13.7 (3.84)	14.7 (3.98)	15.7 (4.38)	17.3 (5.14)	<0.001
Ca	8.30 (0.96)	8.26 (0.97)	8.33 (0.88)	8.26 (0.98)	8.33 (1.01)	0.327
Cl	104 (8.07)	104 (7.52)	105 (7.14)	105 (8.71)	103 (8.64)	<0.001
GLU	155 (86.2)	144 (71.1)	157 (88.4)	156 (91.3)	161 (91.7)	0.001
K	4.21 (0.79)	3.97 (0.64)	4.15 (0.76)	4.27 (0.80)	4.46 (0.87)	<0.001
Na	139 (6.85)	138 (6.23)	139 (5.69)	139 (7.74)	138 (7.40)	<0.001
TCO_2_	24.2 (6.48)	26.3 (6.98)	24.5 (6.03)	23.6 (6.18)	22.3 (6.06)	<0.001
Lac	2.30 (1.79)	1.83 (1.21)	2.13 (1.56)	2.38 (1.84)	2.85 (2.25)	<0.001
Pco_2_	42.2 (12.9)	43.8 (14.6)	42.2 (12.3)	42.1 (12.4)	40.7 (11.9)	0.001
Ph	7.35 (0.10)	7.38 (0.09)	7.36 (0.10)	7.34 (0.10)	7.33 (0.11)	<0.001
Po_2_	116 (96.8)	124 (101)	125 (101)	113 (90.9)	104 (91.9)	<0.001
INR	1.70 (1.10)	1.52 (0.86)	1.63 (1.07)	1.74 (1.03)	1.92 (1.34)	<0.001
PT	18.4 (11.4)	16.6 (8.77)	17.7 (11.9)	18.7 (10.3)	20.7 (13.6)	<0.001
APTT	40.5 (25.0)	37.1 (21.8)	37.7 (21.9)	42.3 (27.6)	44.9 (27.3)	<0.001
ALT	77.0 (361)	50.2 (220)	53.2 (137)	72.7 (336)	132 (581)	0.004
AST	109 (421)	65.3 (372)	71.5 (166)	108 (452)	191 (573)	<0.001
TB	1.89 (4.47)	1.25 (3.29)	1.28 (2.79)	1.89 (4.18)	3.12 (6.44)	<0.001
CRE	1.69 (1.39)	0.85 (0.46)	1.30 (0.68)	1.88 (1.19)	2.75 (1.91)	<0.001
URE	36.4 (27.5)	21.5 (14.1)	29.9 (20.5)	40.2 (26.1)	53.8 (33.9)	<0.001
LDH	335 (310)	232 (94.3)	282 (118)	345 (180)	481 (543)	<0.001
Sa						0.430
No	874 (33.7%)	220 (34.0%)	203 (31.3%)	231 (35.6%)	220 (33.9%)	
Yes	1719 (66.3%)	428 (66.0%)	445 (68.7%)	417 (64.4%)	429 (66.1%)	
Vp						<0.001
No	954 (36.8%)	290 (44.8%)	237 (36.6%)	227 (35.0%)	200 (30.8%)	
Yes	1,639 (63.2%)	358 (55.2%)	411 (63.4%)	421 (65.0%)	449 (69.2%)	
Gc						0.095
No	1746 (67.3%)	448 (69.1%)	453 (69.9%)	429 (66.2%)	416 (64.1%)	
Yes	847 (32.7%)	200 (30.9%)	195 (30.1%)	219 (33.8%)	233 (35.9%)	
ABX						0.750
No	1 (0.04%)	0 (0.00%)	1 (0.15%)	0 (0.00%)	0 (0.00%)	
Yes	2,592 (100.0%)	648 (100%)	647 (99.8%)	648 (100%)	649 (100%)	
Ventilation						0.835
No	349 (13.5%)	84 (13.0%)	84 (13.0%)	87 (13.4%)	94 (14.5%)	
Yes	2,244 (86.5%)	564 (87.0%)	564 (87.0%)	561 (86.6%)	555 (85.5%)	
CRRT						<0.001
No	2,374 (91.6%)	635 (98.0%)	620 (95.7%)	589 (90.9%)	530 (81.7%)	
Yes	219 (8.45%)	13 (2.01%)	28 (4.32%)	59 (9.10%)	119 (18.3%)	
Hosp_day	18.5 (18.4)	18.9 (19.0)	19.0 (19.0)	17.7 (16.5)	18.2 (19.0)	0.511
Icu_day	6.98 (8.43)	7.36 (9.60)	7.47 (8.81)	6.96 (8.32)	6.12 (6.66)	0.005
Death_hosp_28days	416 (16.0%)	59 (9.10%)	83 (12.8%)	84 (13.0%)	190 (29.3%)	<0.001
Death_icu_28days	445 (17.2%)	68 (10.5%)	88 (13.6%)	92 (14.2%)	197 (30.4%)	<0.001

Data are presented as mean (standard deviation, SD) for continuous variables and as *n* (%) for categorical variables. EASIX, Endothelial Activation and Stress Index; AKI, Acute kidney injury; CKD, Chronic kidney disease; HLD, Hyperlipidemia; IHD, Ischemic heart disease; COPD, Chronic obstructive pulmonary disease; SOFA, Sequential organ failure assessment; APS III, Acute physiology score III; SAPS II, Simplified acute physiology score II; OASIS, Oxford acute severity of illness score; APACHE II, Acute physiology and chronic health evaluation II; HR, Heart rate; NBPS, Non-invasive blood pressure systolic; NBPD, Non-invasive blood pressure diastolic; RR, Respiratory rate; SpO₂, Oxygen saturation; HCT, Hematocrit; Hb, Hemoglobin; PLT, Platelet; RDW, Red cell distribution width; RBC, Red blood cell count; WBC, White blood cell count; ALB, Albumin; AG, Anion gap; GLU, Glucose; K, Potassium; Na, Sodium; TCO₂, Total carbon dioxide; PCO₂, Partial pressure of carbon dioxide; LAC, Lactate; PH, Acidity and alkalinity; PO₂, Partial pressure of oxygen; INR, International normalized ratio; PT, Prothrombin time; APTT, Activated partial thromboplastin time; ALT, Alanine aminotransferase; AST, Aspartate aminotransferase; TB, Total bilirubin; CRE, Creatinine; URE, Urea nitrogen; CRRT, Continuous renal replacement therapy; Sa, Analgesic and sedative drugs; Vp, Vasoactive drugs; Gc, Corticosteroids; ABX, Antibiotics.

### Multivariable Cox regression analysis of the association between EASIX and short-term mortality in patients with urosepsis

Multivariable Cox proportional hazards models were used to systematically assess the association between the EASIX score and 28-day ICU and in-hospital mortality in patients with urosepsis (Table 2). For ICU mortality, each unit increase in the EASIX score was associated with an elevated risk: a 17% increase in the unadjusted model (Model 1; HR 1.17, 95% CI 1.14–1.20, *p* < 0.001), a 14% increase after adjusting for demographics and comorbidities (Model 2; HR 1.14, 95% CI 1.11–1.17, *p* < 0.001), and a 12% increase after further adjustment for disease severity scores and laboratory parameters (Model 3; HR 1.12, 95% CI 1.08–1.16, *p* < 0.001). While the per-unit hazard ratio of 1.07–1.17 represents a modest effect size, the clinical utility of EASIX lies in its ability to stratify patients across its full distribution. As shown in Table 1, patients in the highest EASIX quartile (Q4) had a 1.57-fold higher risk of mortality compared to those in the lowest quartile (Q1), providing a more clinically interpretable measure of risk stratification. A similar trend was observed for 28-day in-hospital mortality. When EASIX was analyzed by quartiles, the middle groups (Q2, Q3) showed no significant difference from Q1 after multivariable adjustment. However, the highest-risk group (Q4) demonstrated a 3.24-fold higher risk of ICU mortality in the unadjusted model (HR 3.24, 95% CI 2.46–4.28, *p* < 0.001). This risk remained elevated in the fully adjusted model (Model 3), with the Q4 group having a 1.57-fold higher risk than the Q1 group (HR 1.57, 95% CI 1.09–2.26, *p* = 0.016). The results for in-hospital mortality were consistent, with the Q4 group maintaining a higher risk in the final model (HR 1.46, 95% CI 1.01–2.15, *p* = 0.048). These findings indicate that EASIX is an independent risk factor for 28-day mortality in patients with urosepsis. The elevated mortality risk associated with the highest EASIX quartile (Q4) underscores its important value for prognostic stratification.

**Table 2:** The relationship between EASIX score and 28 day ICU/hospital mortality rate.

Characteristic	Model 1			Model 2			Model 3		
	HR^1^	95% CI^1^	*p*-value	HR^1^	95% CI^1^	*p*-value	HR^1^	95% CI^1^	*p*-value
EASIX	1.17	1.14, 1.20	<0.001	1.14	1.11, 1.17	<0.001	1.12	1.08, 1.16	<0.001
EASIX group									
Q1	Ref	Ref		Ref	Ref		Ref	Ref	
Q2	1.23	0.90, 1.69	0.197	1.06	0.77,1.47	0.728	1.05	0.74, 1.48	0.777
Q3	1.38	1.01, 1.89	0.045	1.05	0.76, 1.47	0.744	0.74	0.50, 1.07	0.111
Q4	3.24	2.46, 4.28	<0.001	2.39	1.77, 3.22	<0.001	1.57	1.09, 2.26	0.016
EASIX	1.17	1.14, 1.20	<0.001	1.16	1.13 1.19	<0.001	1.12	1.08, 1.16	
EASIX group									<0.001
Q1	Ref	Ref		Ref	Ref		Ref	Ref	
Q2	1.36	0.97, 1.89	0.075	1.19	0.85 1.67	0.321	1.13	0.79, 1.62	0.506
Q3	1.47	1.05, 2.05	0.024	1.20	0.85, 1.69	0.306	0.79	0.53, 1.16	0.228
Q4	3.26	2.44, 4.37	<0.001	2.64	1.93, 3.60	<0.001	1.46	1.01, 2.15	0.048

^1^HR, Hazard Ratio; CI, Confidence Interval.

### Subgroup analysis

**Figure 2:**
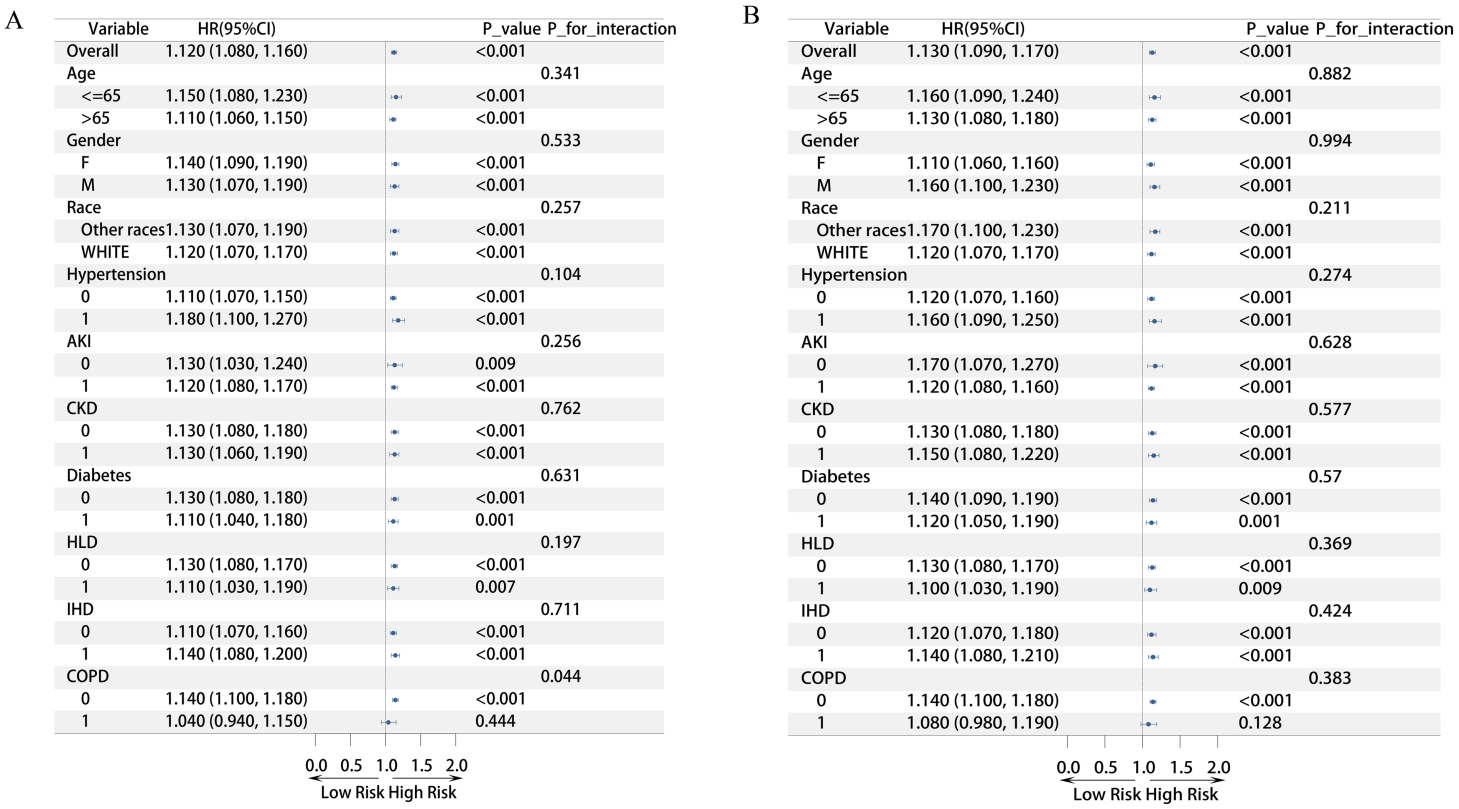
Subgroup analysis of EASIX and 28 day ICU/HOSP mortality rate. **(A)** Subgroup analysis of EASIX and 28 day ICU mortality rate; **(B)** Subgroup analysis of EASIX and 28 day hospital mortality rate.

Subgroup analysis revealed that a higher EASXI score was associated with an increased risk of adverse outcomes (Figure 2A). This association was consistent across most subgroups. For the endpoint of 28-day ICU mortality, the overall hazard ratio (HR) was 1.12 (95% CI: 1.08–1.16, *p* < 0.001), indicating a 12% average increase in ICU mortality risk for each one-unit increase in the EASXI score. In subgroups defined by age, sex, race, hypertension, acute kidney injury (AKI), chronic kidney disease (CKD), diabetes, hyperlipidemia (HLD), and ischemic heart disease (IHD), the hazard ratio was consistently greater than 1 with a 95% confidence interval that did not cross 1, and all subgroup *p*-values were less than 0.05. This consistency further confirms the robustness of the association. Notably, a significant interaction was observed for chronic obstructive pulmonary disease (COPD) (*p* for interaction = 0.044). The EASXI score remained a significant predictor of ICU mortality in patients without COPD (HR = 1.14, *p* < 0.001) but not in those with COPD (HR = 1.04, *p* = 0.444). This suggests that COPD may be an effect modifier, attenuating the predictive power of the EASXI score for ICU mortality risk. For the endpoint of 28-day in-hospital mortality, the overall HR was 1.13 (95% CI: 1.09–1.17, *p* < 0.001), demonstrating that a higher EASXI score was also associated with an increased risk of in-hospital death (Figure 2B). Across all subgroups—including those for age, sex, race, and comorbidities—the hazard ratio was consistently greater than 1 and statistically significant. No significant interactions (with *p* < 0.05) were detected for any of these factors, indicating that the association between the EASXI score and in-hospital mortality is highly consistent across different patient populations and is not significantly modified by the factors examined. These findings underscore the potential value of the EASXI score as a risk assessment tool applicable across diverse populations and provide a basis for individualized risk stratification.

### The incremental effect of EASIX

We used area under the curve (AUC) analysis to evaluate whether adding the EASIX score to established severity-of-illness scores (SOFA, APS III, SAPS II, OASIS, Charlson, APACHE II) improved predictive performance of 28-day ICU mortality. As shown in >Supplementary Figures 1A–F, incorporating EASIX consistently improved the predictive performance of all scores. The AUC increased for SOFA (from 0.64 to 0.67), APS III (0.65 to 0.69), SAPS II (0.65 to 0.69), OASIS (0.57 to 0.67), Charlson (0.57 to 0.66), and APACHE II (0.62 to 0.67).

### External cohort verification

**Figure 3:**
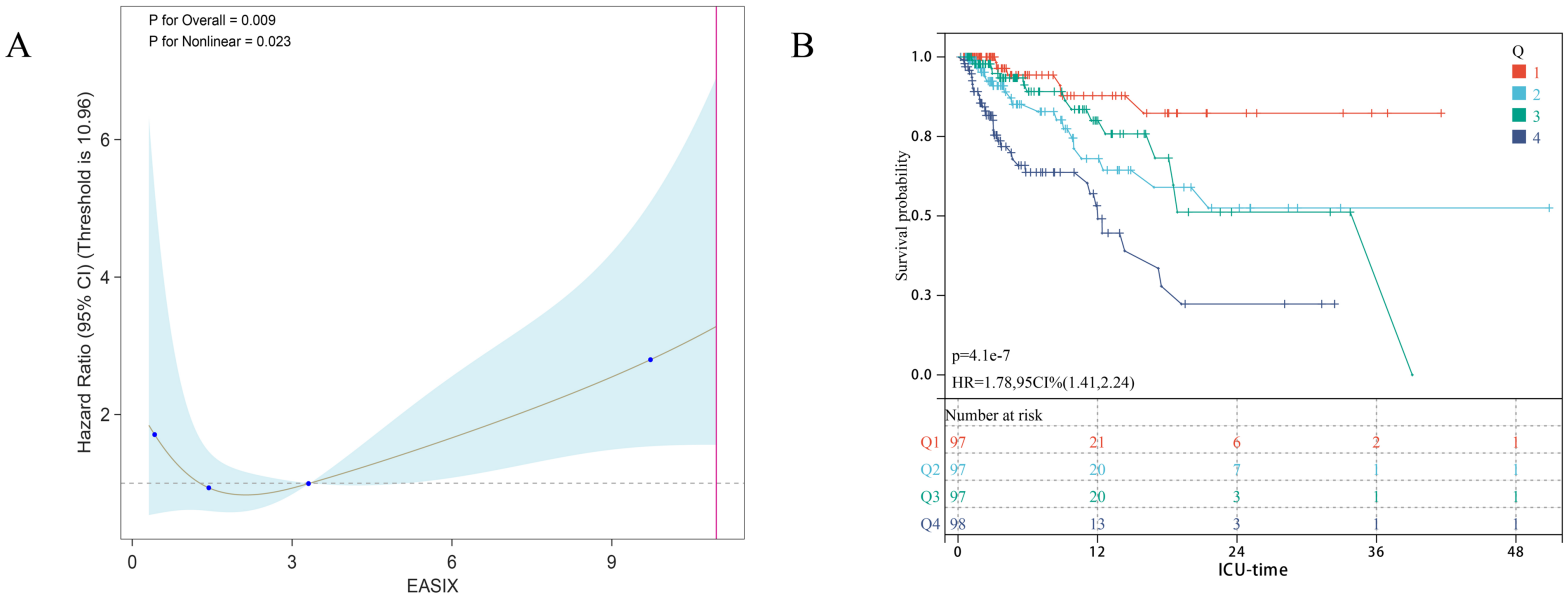
Association between EASIX and 28-day survival in the external validation cohort. **(A)** Restricted cubic spline plot showing the dose–response relationship between continuous EASIX values and 28-day in-hospital mortality in the external validation cohort. **(B)** Kaplan–Meier survival curves illustrate the probability of survival from ICU death 28 days, stratified by EASIX score stratification.

We further assessed the predictive value of the EASIX score for 28-day ICU mortality in an external validation cohort of 389 patients with urosepsis (28-day mortality rate = 19.28%). As shown in Table 3, the EASIX score demonstrated a significant and independent association with 28-day ICU mortality across three statistical models with varying degrees of adjustment for confounding factors. In the unadjusted model (Model 1), EASIX as a continuous variable was associated with a 28% increase in mortality risk per unit increment [Hazard Ratio (HR) = 1.28, 95% CI: 1.15–1.31, *p* < 0.001]. When analyzed by EASIX quartiles, mortality risk increased with each higher quartile, showing a clear dose–response relationship. Specifically, patients in the highest quartile (Q4) had a 7.56-fold greater risk of death compared to those in the lowest quartile (Q1) (HR = 7.56, 95% CI: 3.17–18.04, *p* < 0.001). After adjusting for some confounders in Model 2, the association remained significant, albeit with a slightly attenuated effect (HR per unit = 1.20, 95% CI: 1.13–1.29, *p* = 0.001). In the quartile analysis for Model 2, the risk in Q4 remained substantial (HR = 4.34, 95% CI: 1.69–11.16, *p* = 0.002), while the risks for Q2 and Q3 were no longer statistically significant. This association persisted in the fully adjusted model (Model 3) for EASIX as a continuous variable (HR = 1.19, 95% CI: 1.08–1.31, *p* < 0.001). Crucially, patients in the Q4 quartile continued to face an elevated risk, which was 3.49 times higher than that of the Q1 group (HR = 3.49, 95% CI: 1.14–10.68, *p* = 0.029). A trend test further confirmed a significant dose–response relationship between EASIX and 28-day mortality (*p* for trend < 0.001). Restricted cubic spline (RCS) analysis revealed a linear relationship between the EASIX score and short-term mortality in patients with pulmonary sepsis (Figure 3A). Kaplan–Meier survival curves corroborated these findings, showing that a higher EASIX score was significantly associated with increased 28-day ICU mortality (HR = 1.78, 95% CI: 1.41–2.24; Figure 3B).

**Table 3:** The relationship between EASIX score and external verification cohort 28 day ICU mortality rate.

Characteristic	Model 1			Model 2			Model 3		
	HR^1^	95% CI^1^	*p*-value	HR^1^	95% CI^1^	*p*-value	HR^1^	95% CI^1^	*p*-value
EASIX	1.28	1.15, 1.31	<0.001	1.20	1.13, 1.29	0.001	1.19	1.08, 1.31	<0.001
EASIX group									
Q1	—	—		—	—		—	—	
Q2	3.14	1.25, 7.86	0.015	2.20	0.86, 5.65	0.101	2.01	0.68, 5.88	0.205
Q3	2.39	0.93, 6.16	0.072	1.51	0.56, 4.08	0.416	1.31	0.43, 3.99	0.635
Q4	7.56	3.17, 18.04	<0.001	4.34	1.69, 11.16	0.002	3.49	1.14, 10.68	0.029

^1^HR, Hazard Ratio; CI, Confidence Interval.

### Construction and validation of risk prediction models related to EASIX

**Figure 4:**
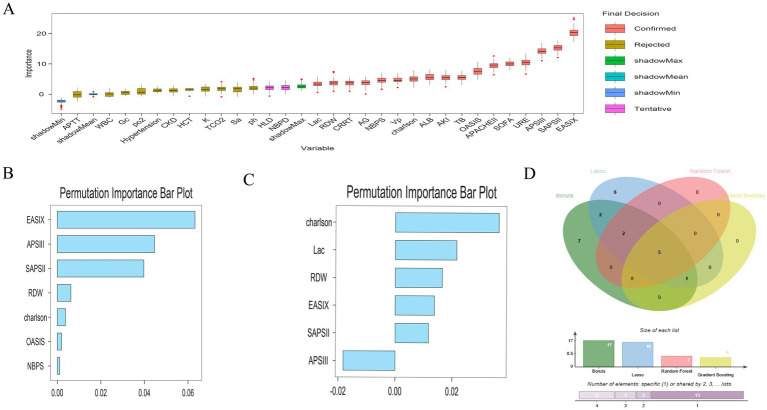
The screening process of feature variables. **(A)**: Boruta feature ranking algorithm, **(B)**: Random Forest; **(C)**: Gradient Boosting; **(D)**: The Venn diagram shows the overlap of candidate predictor variables identified as important in four feature selection methods (Boruta algorithm, LASSO-COX regression random, forest machine learning algorithm and Gradient Boosting learning algorithm).

**Figure 5:**
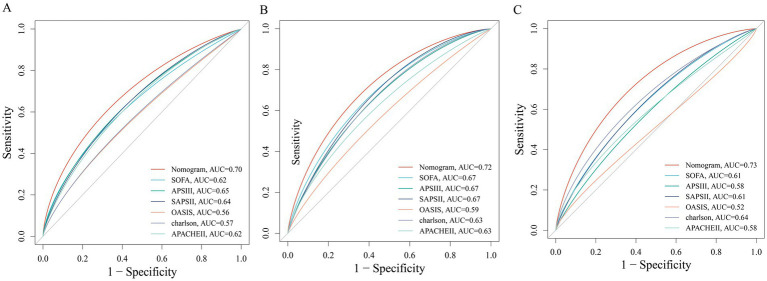
Receiver operating characteristic (ROC) curves comparing the predictive performance of the novel prediction model and conventional severity scores for 28-day mortality. **(A)** test cohort 1; **(B)** train cohort 2; **(C)** external verification cohort.

**Figure 6:**
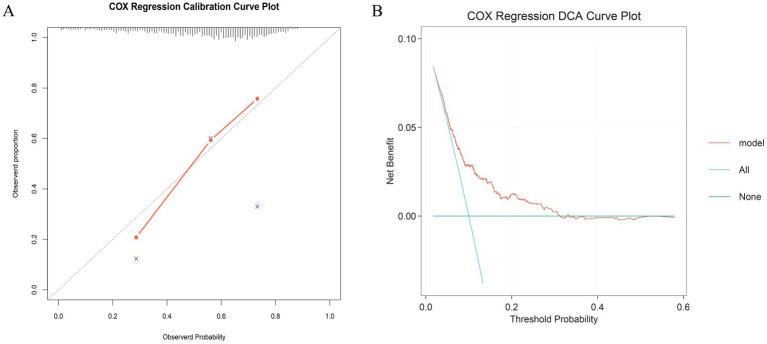
Calibration and decision curve analysis of the prediction model **(A)** Calibration curve; **(B)** DCA curve.

Within the internal test cohort, all patients were randomly assigned to a training set or a test set in a 7:3 ratio. After excluding variables with collinearity (Variance Inflation Factor, VIF > 5; >Supplementary Table S4), we initially applied the Boruta algorithm to screen 30 candidate features for association with the primary endpoint (28-day ICU mortality). Variables in the orange ‘Confirmed’ zone were identified as important features, resulting in the selection of 17 variables associated with 28-day ICU mortality (Figure 4A). Among these, EASIX was identified as the most important feature. Subsequently, we employed Least Absolute Shrinkage and Selection Operator (LASSO) regression on the same 30 candidate variables, which yielded a set of 16 features (>Supplementary Figures S2A,B). We also performed feature selection using two machine learning algorithms: Random Forest, which identified 7 features (Figure 4B), and Gradient Boosting, which identified 6 (Figure 4C). A Venn diagram was used to identify the intersection of the features selected by these four methods, revealing five shared core prognostic variables (Figure 4D). Finally, we performed multivariable Cox regression analysis to definitively identify independent predictors. This process confirmed four independent predictors—EASIX, Charlson Comorbidity Index, Red Cell Distribution Width (RDW), and Simplified Acute Physiology Score II (SAPS II)—which were used to construct a risk prediction model for 28-day ICU mortality in patients with urosepsis (Table 4). Compared to traditional severity scores (SOFA, APS III, SAPS II, OASIS, Charlson, and APACHE II), the prediction model demonstrated improved predictive performance for predicting 28-day ICU mortality, with area under the receiver operating characteristic (AUROC) values of 0.70 in the training cohort (Figure 5A), 0.72 in the validation cohort (Figure 5B), and 0.73 in the external real-world cohort (Figure 5C). The calibration curve showed a modest deviation from the ideal diagonal (Figure 6A). Overall, however, the model demonstrated acceptable calibration, indicating a good agreement between predicted probabilities and observed outcomes across the spectrum of risk. Decision curve analysis confirmed the clinical utility of the prediction model (Figure 6B). Compared to default strategies, the model provided a superior net benefit across a substantial range of threshold probabilities (approximately 20 to 50%).

**Table 4:** Final multivariable Cox proportional hazards model for 28-day mortality.

Variable	Coefficient (β)	Hazard ratio (HR)	Standard error (SE)	Z-value	*p*-value
EASIX	0.110	1.116	0.016	7.007	<0.001
SAPSII	0.021	1.022	0.004	5.164	<0.001
Charlson	0.077	1.080	0.020	3.816	<0.001
RDW	0.092	1.097	0.020	4.590	<0.001

## Discussion

To our knowledge, this study is the first to establish, within a large cohort of critically ill patients with urosepsis, that the EASIX score is significantly and independently associated with short-term mortality. We acknowledge that EASIX has been extensively investigated in other critical illness settings, including cardiovascular disease, severe asthma, and sepsis populations more broadly; however, its prognostic value in the specific context of urosepsis has not been previously reported. We observed a clear gradient of increasing disease severity, organ dysfunction, and mortality risk with rising EASIX levels. This dose–response relationship was consistently validated by restricted cubic spline analysis and in multivariable-adjusted models. Notably, even after comprehensive adjustment for confounders, patients in the highest EASIX quartile (Q4) had a 1.57-fold higher risk of 28-day ICU mortality compared to those in the lowest quartile, underscoring the unique value of EASIX in identifying high-risk patients. Furthermore, by integrating multiple advanced machine learning algorithms—including Boruta, LASSO regression, Random Forest, and Gradient Boosting—with traditional statistics, we innovatively identified four core predictors: EASIX, the Charlson Comorbidity Index, Red Cell Distribution Width (RDW), and the Simplified Acute Physiology Score II (SAPS II). These were used to construct a practical prediction model. This model demonstrated superior predictive performance compared to traditional scoring systems (such as SOFA and APACHE II), achieving area under the curve (AUC) values of 0.70 in the training cohort, 0.72 in the validation cohort, and 0.73 in the external real-world cohort. More importantly, decision curve analysis confirmed the model’s robust clinical utility, demonstrating a significant net benefit across a wide range of threshold probabilities (approximately 20 to 50%). It is particularly noteworthy that incorporating EASIX significantly enhanced the predictive capability of all commonly used critical illness scores, with AUC improvements ranging from 0.03 to 0.10. This indicates that EASIX provides substantial incremental value by contributing prognostic information independent of existing assessment systems.

Subgroup analysis further reinforced the reliability of EASIX as a risk assessment tool. Its predictive power for mortality remained consistent across subgroups defined by age, sex, race, and various comorbidities, indicating broad applicability. However, a notable effect modification was observed in patients with COPD. The association between EASIX and ICU mortality was not significant in patients with COPD (HR = 1.04, *p* = 0.444) but remained strong in those without COPD (HR = 1.14, *p* < 0.001). This phenomenon may be linked to the distinct pathophysiological state in COPD, including chronic inflammation, oxidative stress, endothelial dysfunction, and vascular remodeling. These underlying conditions may alter the host response to the acute insult of sepsis, potentially modulating the relationship captured by EASIX. This important finding suggests that individualized risk stratification thresholds may be necessary for patients with different underlying conditions. It also provides a crucial direction for future research into the context-specific applicability of EASIX.

Urosepsis, a severe complication of urinary tract infections, has seen a rising incidence in recent years. This trend is closely linked to the increasing prevalence of urological diseases and procedures, as well as growing antimicrobial resistance, posing a significant challenge to both patients and public health systems (4, 5). Identifying reliable and clinically applicable biomarkers is crucial for improving outcomes in these patients. As a composite marker derived from parameters that become abnormal following endothelial activation and organ injury, an elevated EASIX score reflects the cumulative burden of sepsis-associated endothelial and microvascular dysfunction. Specifically, decreased platelet counts and hemoglobin suggest endothelial-related consumptive coagulopathy and microangiopathic hemolysis; elevated liver enzymes (ALT, AST), bilirubin, lactate, urea, and creatinine levels reflect concomitant hepatic/renal impairment and tissue hypoperfusion; and abnormal coagulation parameters (INR, PT, APTT) indicate coagulation system activation secondary to endothelial disturbance. These changes align closely with the core pathophysiology of sepsis—endothelial dysfunction (20, 21). During sepsis, pathogens and their toxins can trigger a “cytokine storm,” leading to endothelial cell activation, apoptosis, and loss of barrier integrity. This, in turn, causes microcirculatory dysfunction, tissue edema, coagulopathy, and ultimately multiple organ failure (22, 23). Notably, the key components of the EASIX formula (LDH, creatinine, platelets) directly correspond to these pathological processes: LDH serves as a marker of cellular damage and hemolysis; creatinine reflects renal function (a system highly vulnerable to microcirculatory impairment in sepsis); and thrombocytopenia is a common manifestation of sepsis-induced coagulopathy and endothelial consumption (24, 25). The multiplicative combination of these parameters—with LDH and platelet count typically contributing larger dynamic ranges—was originally proposed by Luft et al. (5) based on their independent associations with endothelial complications. While the formula does not mathematically equalize the relative contribution of each component, this weighting is clinically appropriate in the context of sepsis, where LDH serves as a highly sensitive marker of acute cellular injury and platelet count captures the consumptive coagulopathy that defines sepsis-associated microangiopathy. In our study, the superior predictive performance of the composite EASIX score compared to its individual components (as shown in the incremental AUC analyses; >Supplementary Figure S1) further supports the construct validity of this formula in patients with urosepsis. Therefore, as a composite metric, EASIX effectively integrates multiple downstream effects of endothelial injury, providing a systematic and quantitative assessment of the severity of systemic endothelial dysfunction. In recent years, this index has been widely used for prognostic assessment in various conditions, including stroke, asthma, malignancies, and cardiovascular diseases (17, 26, 27). Consistent with this body of evidence, our study is the first to demonstrate that an elevated EASIX score, reflecting the severity of endothelial injury and multiorgan dysfunction, is an independent prognostic factor for both 28-day ICU mortality and in-hospital all-cause mortality in patients with established urosepsis.

This study has several limitations. First, its retrospective and observational design inherently carries a risk of residual confounding and unmeasured bias, despite statistical adjustments. While we adjusted for disease severity using SOFA, APACHE II, and SAPS II scores, we did not explicitly categorize patients by the presence of septic shock—a more advanced stage of sepsis with well-established independent prognostic value. Although SOFA scores capture the degree of organ dysfunction and were included in our models, we acknowledge that the absence of septic shock as a separate covariate may result in residual confounding. Additionally, while we adjusted for the presence of comorbidities such as chronic kidney disease (CKD) and hyperlipidemia in Model 2, we did not have sufficient granular data on the severity or stage of these conditions (e.g., CKD stage based on eGFR, or the extent of liver dysfunction). As the components of EASIX—particularly creatinine and LDH—can be significantly influenced by the severity of underlying chronic diseases, the lack of adjustment for disease stage may introduce residual confounding. This represents an important limitation of our study. Future prospective studies should incorporate detailed comorbidity staging to more precisely estimate the independent prognostic value of EASIX. Second, the primary cohort was sourced solely from the MIMIC-IV database, which reflects patient data from specific U. S. medical centers; this may limit the generalizability of our findings across different healthcare systems, ethnic populations, and clinical practices. Although we validated the model using an external cohort, which supported its robustness, this cohort was relatively small and featured a different case mix, underscoring the need for validation in larger, more diverse prospective cohorts. Third, our analysis of EASIX relied on a single baseline measurement obtained within the first 24 h of ICU admission. While this time window is commonly used in retrospective database studies to ensure data completeness and consistency with routine clinical sampling, we acknowledge that sepsis is a rapidly evolving condition in which endothelial injury and organ function may change substantially within hours. Consequently, measurements taken at 24 h may not fully capture the earliest pathophysiological changes that occur immediately upon ICU arrival, and a one-time measurement—regardless of timing—cannot reflect the dynamic fluctuations in risk over time. Future research should investigate the value of serial EASIX measurements, ideally with earlier time points (e.g., within 6 h of admission), to enable more precise dynamic risk assessment and monitoring of treatment response. Additionally, the components of EASIX—particularly LDH and platelet count—are not specific to sepsis and can be elevated or depressed in various chronic conditions, including chronic kidney disease, heart failure, and liver dysfunction. As patients in higher EASIX quartiles had a greater burden of such comorbidities, the prognostic value of EASIX observed in this study may partly reflect the severity of underlying chronic diseases rather than solely capturing sepsis-related endothelial injury. While we adjusted for the presence of these comorbidities in our multivariable models, we cannot fully exclude residual confounding due to unmeasured disease severity or chronicity. This limitation underscores the need for future studies incorporating more specific endothelial biomarkers to complement EASIX in the setting of sepsis.

Fourth, although we integrated multiple machine learning algorithms to select core variables, the final model’s performance was moderate (AUROC 0.70–0.73), indicating that the prognosis of urosepsis is influenced by highly complex factors not fully captured by our current model. Furthermore, while the prediction model demonstrated good statistical calibration and clinical utility, its practical usability in real-world clinical settings and actual impact on decision-making require further validation through prospective interventional studies. Therefore, future work should focus on multi-center prospective studies and, ultimately, interventional research that integrates EASIX into clinical workflows to empirically test its value in improving patient outcomes. Concurrently, exploring the relationship between EASIX and established sepsis-specific endothelial biomarkers (e.g., von Willebrand factor, syndecan-1) could deepen the mechanistic understanding of its biological underpinnings. Fifth, additionally, while we demonstrated that EASIX provides incremental prognostic value beyond conventional severity scores (SOFA, APACHE II, etc.), we did not directly compare EASIX with other established biomarkers such as lactate, procalcitonin (PCT), neutrophil-to-lymphocyte ratio (NLR), or platelet-to-lymphocyte ratio (PLR). In our machine learning feature selection analyses, these specific biomarkers were not identified as core prognostic factors for 28-day mortality in urosepsis; however, direct head-to-head comparisons would be valuable to further establish the relative performance of EASIX. Additionally, our fully adjusted model (Model 3) included variables that may act as mediators on the causal pathway between EASIX and mortality, such as severity scores (SOFA, APACHE II) and laboratory parameters (lactate, creatinine). Adjusting for these mediators may result in overadjustment bias and could attenuate the true effect size of EASIX. Future prospective studies should include these biomarkers to better define the incremental value of EASIX in the context of existing prognostic tools.

## Conclusion

Our study strongly establishes EASIX as a simple, readily available, and reliable prognostic marker for urosepsis. By providing a practical prognostic risk-stratification tool for patients with urosepsis admitted to the ICU, our work paves the way for future research to investigate whether targeted interventions, guided by this assessment, can ultimately improve survival in this vulnerable patient population.

## Data Availability

The datasets presented in this study can be found in online repositories. The names of the repository/repositories and accession number(s) can be found at: https://pediatrixlab.com/MIMIC.html.
