# Supplementary material for: Relationship of the Endothelial Activation and Stress Index with 28-day mortality in urosepsis patients: a retrospective two-cohort investigation
Source: Front Med (Lausanne). 2026 May 12;13:1761104. doi: 10.3389/fmed.2026.1761104 (PMC13201423; doi:10.3389/fmed.2026.1761104)
Supplement: Supplementary file 1 [file Supplementary_file_1.pdf]

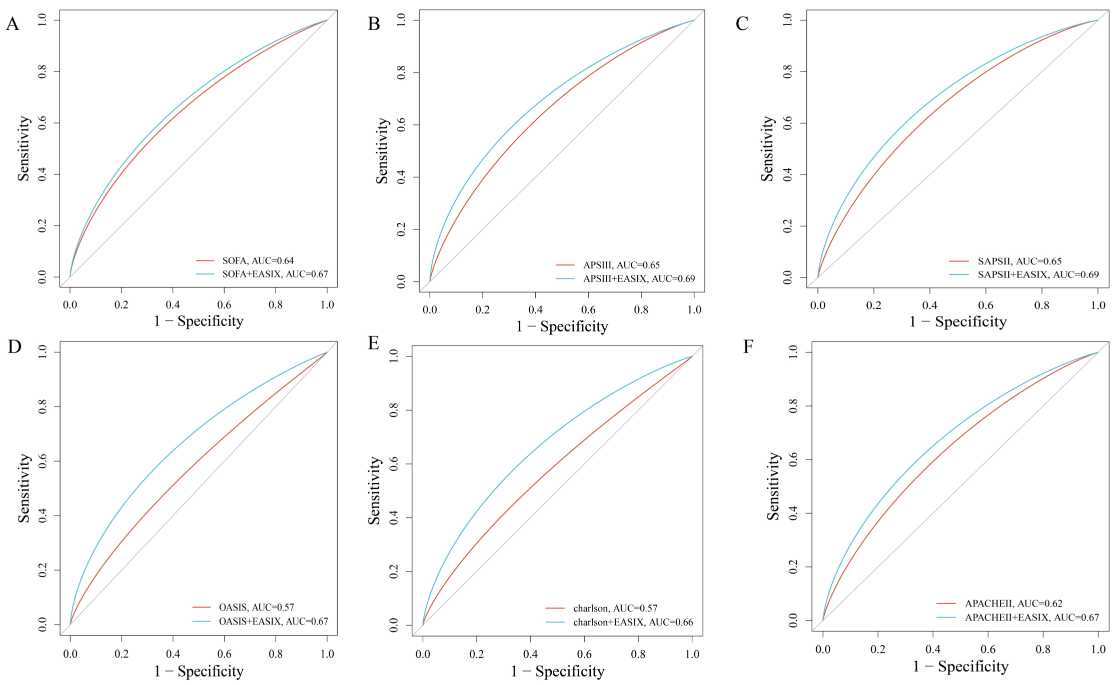

Figure S1: Incremental prognostic value of adding EASIX to conventional severity scores for predicting 28-day in-ICU mortality.

Note: (A-F) Receiver operating characteristic (ROC) curves demonstrate the improvement in predictive performance when the EASIX is added to five conventional severity scores: (A) SOFA, (B) APS III, (C) SAPS II, (D) OASIS, (E) charlson and (F) APACHE II. Each panel compares the AUC of the original severity score (red) against the combined model of the severity score plus EASXI (blue) in the internal cohort. The specific AUC values for each model are provided in the main text.

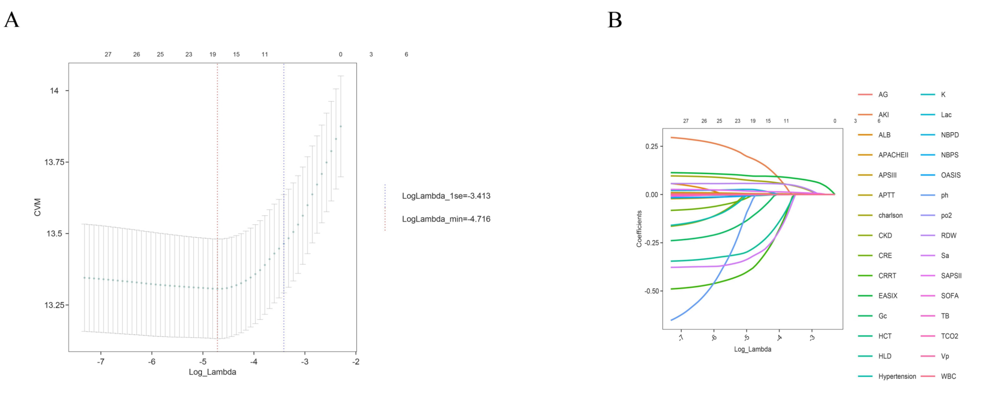

Figure S2: Variable selection using the Least Absolute Shrinkage and Selection Operator (LASSO) regression.

Note: (A) LASSO coefficient profiles of the candidate prognostic variables. Each curve represents

the coefficient path of a variable as the penalty parameter (λ) increases.(B) Ten-fold cross-validation for tuning parameter (λ) selection in the LASSO model. The left vertical dashed line (lambda.min) is drawn at the value of λ that gives the minimum mean cross-validated error. The right vertical dashed line (lambda.1se) is drawn at the largest value of λ such that the error is within one standard error of the minimum, which was used for feature selection.

TableS1: Summary descriptives table by groups of 28day in-hospital mortality rate

	[ALL]	Survivor	No-survivor	P-value
	N=2593	*N=2148*	*N=445*	
Q:				<0.001
Q1	648 (25.0%)	580 (27.0%)	68 (15.3%)	
Q2	648 (25.0%)	560 (26.1%)	88 (19.8%)	
Q3	648 (25.0%)	556 (25.9%)	92 (20.7%)	
Q4	649 (25.0%)	452 (21.0%)	197 (44.3%)	
EASIX	3.21 (2.92)	2.89 (2.63)	4.72 (3.70)	<0.001
Age	70.9 (15.0)	70.5 (15.0)	72.5 (14.8)	0.013
Gender:				0.412
F	1476 (56.9%)	1231 (57.3%)	245 (55.1%)	
M	1117 (43.1%)	917 (42.7%)	200 (44.9%)	
Race:				0.055
Other races	880 (33.9%)	711 (33.1%)	169 (38.0%)	
WHITE	1713 (66.1%)	1437 (66.9%)	276 (62.0%)	
Weight	82.5 (28.1)	82.7 (28.6)	81.2 (25.3)	0.244
Hypertension:				0.001
No	1689 (65.1%)	1368 (63.7%)	321 (72.1%)	
Yes	904 (34.9%)	780 (36.3%)	124 (27.9%)	
AKI:				<0.001

	[ALL]	Survivor	No-survivor	P-value
	N=2593	*N=2148*	*N=445*	
No	981 (37.8%)	885 (41.2%)	96 (21.6%)	
Yes	1612 (62.2%)	1263 (58.8%)	349 (78.4%)	
CKD:				0.037
No	1817 (70.1%)	1524 (70.9%)	293 (65.8%)	
Yes	776 (29.9%)	624 (29.1%)	152 (34.2%)	
Diabetes:				0.555
No	1620 (62.5%)	1336 (62.2%)	284 (63.8%)	
Yes	973 (37.5%)	812 (37.8%)	161 (36.2%)	
HLD:				0.001
No	1659 (64.0%)	1342 (62.5%)	317 (71.2%)	
Yes	934 (36.0%)	806 (37.5%)	128 (28.8%)	
IHD:				0.802
No	1580 (60.9%)	1306 (60.8%)	274 (61.6%)	
Yes	1013 (39.1%)	842 (39.2%)	171 (38.4%)	
COPD:				0.530
No	2139 (82.5%)	1777 (82.7%)	362 (81.3%)	
Yes	454 (17.5%)	371 (17.3%)	83 (18.7%)	
SOFA	6.62 (3.22)	6.32 (3.04)	8.09 (3.64)	<0.001
APSIII	57.8 (20.2)	55.7 (19.0)	67.7 (22.6)	<0.001
SAPSII	44.5 (13.2)	43.2 (12.6)	50.7 (14.2)	<0.001
OASIS	35.7 (8.25)	35.3 (8.06)	37.6 (8.90)	<0.001
charlson	6.37 (2.87)	6.20 (2.82)	7.18 (2.97)	<0.001
APACHEII	21.6 (6.72)	21.0 (6.48)	24.2 (7.25)	<0.001
HR	91.8 (21.3)	91.7 (21.2)	92.5 (21.4)	0.459
NBPS	120 (25.5)	121 (25.4)	116 (25.6)	<0.001

	[ALL]	Survivor	No-survivor	P-value
	N=2593	*N=2148*	*N=445*	
NBPD	67.8 (20.1)	68.2 (20.0)	66.0 (20.7)	0.044
RR	20.2 (6.45)	20.2 (6.44)	20.2 (6.52)	0.913
Spo2	96.5 (4.75)	96.5 (4.83)	96.3 (4.33)	0.431
temperaturef	98.0 (4.27)	98.0 (4.43)	97.8 (3.35)	0.280
HCT	31.3 (6.61)	31.4 (6.55)	30.7 (6.89)	0.051
HB	10.1 (2.19)	10.1 (2.17)	9.89 (2.25)	0.038
PLT	210 (117)	213 (117)	193 (113)	0.001
RDW	16.1 (2.54)	15.9 (2.47)	16.8 (2.76)	<0.001
RBC	3.40 (0.78)	3.42 (0.77)	3.30 (0.81)	0.004
WBC	13.9 (15.1)	13.6 (14.3)	15.4 (18.7)	0.027
ALB	2.90 (0.62)	2.92 (0.60)	2.81 (0.71)	0.008
AG	15.3 (4.55)	15.2 (4.48)	16.0 (4.87)	0.003
Ca	8.30 (0.96)	8.29 (0.95)	8.31 (1.01)	0.777
Cl	104 (8.07)	104 (7.93)	103 (8.71)	0.176
GLU	155 (86.2)	154 (85.7)	157 (88.6)	0.458
K	4.21 (0.79)	4.19 (0.78)	4.34 (0.85)	<0.001
Na	139 (6.85)	139 (6.75)	138 (7.29)	0.468
TCO2	24.2 (6.48)	24.4 (6.48)	23.2 (6.44)	0.001
Lac	2.30 (1.79)	2.22 (1.74)	2.65 (2.00)	<0.001
Pco2	42.2 (12.9)	42.3 (12.9)	41.8 (13.0)	0.460
ph	7.35 (0.10)	7.36 (0.10)	7.34 (0.11)	0.002
po2	116 (96.8)	119 (99.4)	104 (82.0)	<0.001
INR	1.70 (1.10)	1.66 (1.05)	1.90 (1.29)	<0.001
PT	18.4 (11.4)	18.0 (10.9)	20.5 (13.4)	<0.001
APTT	40.5 (25.0)	39.8 (24.6)	43.6 (26.5)	0.006

	[ALL]	Survivor	No-survivor	P-value
	N=2593	*N=2148*	*N=445*	
ALT	77.0 (361)	77.3 (387)	75.2 (195)	0.861
AST	109 (421)	108 (452)	111 (217)	0.835
TB	1.89 (4.47)	1.66 (3.94)	3.00 (6.31)	<0.001
CRE	1.69 (1.39)	1.64 (1.36)	1.94 (1.50)	<0.001
URE	36.4 (27.5)	34.5 (25.9)	45.7 (33.0)	<0.001
LDH	335 (310)	322 (315)	400 (280)	<0.001
Sa:				<0.001
No	874 (33.7%)	764 (35.6%)	110 (24.7%)	
Yes	1719 (66.3%)	1384 (64.4%)	335 (75.3%)	
Vp:				<0.001
No	954 (36.8%)	858 (39.9%)	96 (21.6%)	
Yes	1639 (63.2%)	1290 (60.1%)	349 (78.4%)	
Gc:				0.044
No	1746 (67.3%)	1465 (68.2%)	281 (63.1%)	
Yes	847 (32.7%)	683 (31.8%)	164 (36.9%)	
ABX:				1.000
No	1 (0.04%)	1 (0.05%)	0 (0.00%)	
Yes	2592 (100.0%)	2147 (100.0%)	445 (100%)	
Ventilation:				0.582
No	349 (13.5%)	285 (13.3%)	64 (14.4%)	
Yes	2244 (86.5%)	1863 (86.7%)	381 (85.6%)	
CRRT:				<0.001
No	2374 (91.6%)	1998 (93.0%)	376 (84.5%)	
Yes	219 (8.45%)	150 (6.98%)	69 (15.5%)	

Table S2: Summary descriptives table by groups of 28day in-hospital mortality rate

	[ALL]	Survivor	No-survivor	P-value
	N=2593	*N=2177*	*N=416*	
EASIX.group:				<0.001
Q1	648 (25.0%)	589 (27.1%)	59 (14.2%)	
Q2	648 (25.0%)	565 (26.0%)	83 (20.0%)	
Q3	648 (25.0%)	564 (25.9%)	84 (20.2%)	
Q4	649 (25.0%)	459 (21.1%)	190 (45.7%)	
EASIX	3.21 (2.92)	2.88 (2.59)	4.93 (3.83)	<0.001
Age	70.9 (15.0)	70.5 (15.0)	72.8 (14.7)	0.003
Gender:				0.184
F	1476 (56.9%)	1252 (57.5%)	224 (53.8%)	
M	1117 (43.1%)	925 (42.5%)	192 (46.2%)	
Race:				0.050
Other races	880 (33.9%)	721 (33.1%)	159 (38.2%)	
WHITE	1713 (66.1%)	1456 (66.9%)	257 (61.8%)	
Weight	82.5 (28.1)	82.7 (28.6)	81.2 (25.3)	0.279
Hypertension:				0.001
No	1689 (65.1%)	1388 (63.8%)	301 (72.4%)	
Yes	904 (34.9%)	789 (36.2%)	115 (27.6%)	
AKI:				<0.001
No	981 (37.8%)	889 (40.8%)	92 (22.1%)	
Yes	1612 (62.2%)	1288 (59.2%)	324 (77.9%)	
CKD:				0.014
No	1817 (70.1%)	1547 (71.1%)	270 (64.9%)	

	[ALL]	Survivor	No-survivor	P-value
	N=2593	*N=2177*	*N=416*	
Yes	776 (29.9%)	630 (28.9%)	146 (35.1%)	
Diabetes:				0.466
No	1620 (62.5%)	1353 (62.1%)	267 (64.2%)	
Yes	973 (37.5%)	824 (37.9%)	149 (35.8%)	
HLD:				0.005
No	1659 (64.0%)	1367 (62.8%)	292 (70.2%)	
Yes	934 (36.0%)	810 (37.2%)	124 (29.8%)	
IHD:				0.585
No	1580 (60.9%)	1332 (61.2%)	248 (59.6%)	
Yes	1013 (39.1%)	845 (38.8%)	168 (40.4%)	
COPD:				0.425
No	2139 (82.5%)	1802 (82.8%)	337 (81.0%)	
Yes	454 (17.5%)	375 (17.2%)	79 (19.0%)	
SOFA	6.62 (3.22)	6.33 (3.05)	8.16 (3.62)	<0.001
APSIII	57.8 (20.2)	55.9 (19.0)	67.7 (22.9)	<0.001
SAPSII	44.5 (13.2)	43.2 (12.6)	50.9 (14.2)	<0.001
OASIS	35.7 (8.25)	35.3 (8.06)	37.8 (8.93)	<0.001
charlson	6.37 (2.87)	6.21 (2.83)	7.20 (2.96)	<0.001
APACHEII	21.6 (6.72)	21.1 (6.50)	24.2 (7.24)	<0.001
HR	91.8 (21.3)	91.7 (21.3)	92.2 (21.2)	0.647
NBPS	120 (25.5)	121 (25.4)	116 (25.6)	<0.001
NBPD	67.8 (20.1)	68.2 (20.1)	65.8 (20.1)	0.025
RR	20.2 (6.45)	20.2 (6.41)	20.3 (6.65)	0.708
Spo2	96.5 (4.75)	96.5 (4.84)	96.3 (4.22)	0.334
temperaturef	98.0 (4.27)	98.0 (4.41)	97.8 (3.44)	0.247

	[ALL]	Survivor	No-survivor	P-value
	N=2593	*N=2177*	*N=416*	
HCT	31.3 (6.61)	31.3 (6.54)	31.0 (6.98)	0.359
HB	10.1 (2.19)	10.1 (2.17)	9.97 (2.29)	0.228
PLT	210 (117)	212 (117)	195 (113)	0.006
RDW	16.1 (2.54)	16.0 (2.51)	16.7 (2.61)	<0.001
RBC	3.40 (0.78)	3.42 (0.77)	3.34 (0.82)	0.074
WBC	13.9 (15.1)	13.6 (14.8)	15.4 (16.6)	0.049
ALB	2.90 (0.62)	2.92 (0.61)	2.80 (0.69)	0.003
AG	15.3 (4.55)	15.2 (4.47)	16.1 (4.91)	<0.001
Ca	8.30 (0.96)	8.30 (0.96)	8.29 (0.97)	0.900
Cl	104 (8.07)	104 (7.93)	103 (8.76)	0.118
GLU	155 (86.2)	154 (85.4)	158 (90.3)	0.337
K	4.21 (0.79)	4.19 (0.78)	4.36 (0.86)	<0.001
Na	139 (6.85)	139 (6.73)	138 (7.42)	0.590
TCO2	24.2 (6.48)	24.4 (6.47)	23.1 (6.45)	<0.001
Lac	2.30 (1.79)	2.22 (1.71)	2.70 (2.12)	<0.001
Pco2	42.2 (12.9)	42.3 (12.9)	41.9 (13.0)	0.569
ph	7.35 (0.10)	7.36 (0.10)	7.34 (0.11)	<0.001
po2	116 (96.8)	119 (99.6)	101 (79.1)	<0.001
INR	1.70 (1.10)	1.67 (1.06)	1.88 (1.30)	0.003
PT	18.4 (11.4)	18.1 (10.9)	20.2 (13.5)	0.003
APTT	40.5 (25.0)	39.9 (24.7)	43.5 (26.3)	0.009
ALT	77.0 (361)	75.4 (382)	85.0 (224)	0.484
AST	109 (421)	105 (441)	127 (291)	0.206
TB	1.89 (4.47)	1.71 (4.11)	2.81 (5.89)	<0.001
CRE	1.69 (1.39)	1.63 (1.35)	2.01 (1.57)	<0.001

	[ALL]	Survivor	No-survivor	P-value
	N=2593	*N=2177*	*N=416*	
URE	36.4 (27.5)	34.4 (25.8)	46.6 (33.4)	<0.001
LDH	335 (310)	320 (310)	415 (298)	<0.001
Sa:				0.001
No	874 (33.7%)	764 (35.1%)	110 (26.4%)	
Yes	1719 (66.3%)	1413 (64.9%)	306 (73.6%)	
Vp:				<0.001
No	954 (36.8%)	862 (39.6%)	92 (22.1%)	
Yes	1639 (63.2%)	1315 (60.4%)	324 (77.9%)	
Gc:				0.058
No	1746 (67.3%)	1483 (68.1%)	263 (63.2%)	
Yes	847 (32.7%)	694 (31.9%)	153 (36.8%)	
ABX:				1.000
No	1 (0.04%)	1 (0.05%)	0 (0.00%)	
Yes	2592 (100.0%)	2176 (100.0%)	416 (100%)	
Ventilation:				0.694
No	349 (13.5%)	290 (13.3%)	59 (14.2%)	
Yes	2244 (86.5%)	1887 (86.7%)	357 (85.8%)	
CRRT:				<0.001
No	2374 (91.6%)	2021 (92.8%)	353 (84.9%)	
Yes	219 (8.45%)	156 (7.17%)	63 (15.1%)	

TableS3: Summary descriptives table by groups of external verification cohort Dead

	[ALL]	Survivor	No-survivor	P-value
	N=389	*N=314*	*N=75*	

	[ALL]	Survivor	No-survivor	P-value
	N=389	*N=314*	*N=75*	
Q:				<0.001
Q1	97 (24.9%)	91 (29.0%)	6 (8.00%)	
Q2	97 (24.9%)	78 (24.8%)	19 (25.3%)	
Q3	97 (24.9%)	82 (26.1%)	15 (20.0%)	
Q4	98 (25.2%)	63 (20.1%)	35 (46.7%)	
EASIX	3.24 (2.89)	2.84 (2.57)	4.87 (3.51)	<0.001
Age	71.3 (14.9)	70.2 (15.0)	76.0 (13.7)	0.001
Gender:				0.930
F	217 (55.8%)	176 (56.1%)	41 (54.7%)	
M	172 (44.2%)	138 (43.9%)	34 (45.3%)	
Race:				0.296
Other races	138 (35.5%)	107 (34.1%)	31 (41.3%)	
WHITE	251 (64.5%)	207 (65.9%)	44 (58.7%)	
BMI	27.8 (4.65)	28.0 (4.58)	26.9 (4.85)	0.068
Hypertension:				0.031
No	257 (66.1%)	199 (63.4%)	58 (77.3%)	
Yes	132 (33.9%)	115 (36.6%)	17 (22.7%)	
AKI:				<0.001
No	151 (38.8%)	140 (44.6%)	11 (14.7%)	
Yes	238 (61.2%)	174 (55.4%)	64 (85.3%)	
CKD:				0.245
No	273 (70.2%)	225 (71.7%)	48 (64.0%)	
Yes	116 (29.8%)	89 (28.3%)	27 (36.0%)	
Diabetes:				0.903
No	244 (62.7%)	196 (62.4%)	48 (64.0%)	

	[ALL] *N=389*	Survivor *N=314*	No-survivor *N=75*	P-value
Yes	145 (37.3%)	118 (37.6%)	27 (36.0%)	
HLD:				0.887
No	254 (65.3%)	204 (65.0%)	50 (66.7%)	
Yes	135 (34.7%)	110 (35.0%)	25 (33.3%)	
IHD:				0.832
No	237 (60.9%)	190 (60.5%)	47 (62.7%)	
Yes	152 (39.1%)	124 (39.5%)	28 (37.3%)	
COPD:				0.411
No	322 (82.8%)	257 (81.8%)	65 (86.7%)	
Yes	67 (17.2%)	57 (18.2%)	10 (13.3%)	
APSIII	57.7 (20.7)	56.4 (20.2)	63.1 (22.2)	0.019
SAPSII	44.6 (13.1)	43.5 (12.7)	49.5 (13.8)	0.001
OASIS	35.2 (8.54)	35.1 (8.19)	35.8 (9.90)	0.590
charlson	6.37 (2.84)	6.07 (2.71)	7.64 (3.05)	<0.001
APACHEII	21.3 (6.81)	20.8 (6.55)	23.1 (7.56)	0.017
HR	90.9 (19.9)	91.3 (19.7)	89.1 (20.9)	0.417
NBPS	120 (25.7)	121 (25.1)	118 (28.4)	0.396
NBPD	67.9 (19.7)	68.3 (18.9)	66.4 (22.7)	0.506
RR	19.8 (5.69)	20.1 (5.82)	18.4 (4.91)	0.009
Spo2	96.6 (3.96)	96.6 (4.04)	96.6 (3.62)	0.944
temperaturef	97.9 (5.15)	97.9 (5.71)	98.0 (1.24)	0.699
HCT	31.3 (6.66)	31.6 (6.60)	30.2 (6.86)	0.111
HB	10.1 (2.19)	10.2 (2.17)	9.72 (2.23)	0.095
PLT	200 (103)	203 (103)	186 (103)	0.185
RDW	16.1 (2.55)	15.9 (2.52)	16.8 (2.57)	0.009

	[ALL]	Survivor	No-survivor	P-value
	N=389	*N=314*	*N=75*	
RBC	3.41 (0.76)	3.43 (0.75)	3.29 (0.78)	0.144
WBC	12.9 (6.95)	12.9 (6.99)	13.0 (6.84)	0.934
ALB	2.90 (0.62)	2.92 (0.61)	2.82 (0.66)	0.304
AG	15.2 (4.33)	15.2 (4.31)	15.6 (4.42)	0.476
Ca	8.28 (0.91)	8.30 (0.88)	8.19 (1.01)	0.386
Cl	104 (8.02)	104 (7.81)	105 (8.87)	0.449
GLU	156 (79.8)	157 (82.0)	151 (70.5)	0.534
K	4.23 (0.81)	4.21 (0.83)	4.31 (0.69)	0.310
Na	139 (6.72)	138 (6.64)	139 (7.02)	0.334
TCO2	24.3 (6.15)	24.5 (6.23)	23.5 (5.78)	0.209
Lac	2.28 (1.67)	2.21 (1.66)	2.57 (1.69)	0.099
Pco2	41.6 (11.7)	41.9 (12.2)	40.3 (9.40)	0.223
ph	7.36 (0.10)	7.36 (0.10)	7.36 (0.11)	0.649
po2	118 (102)	121 (105)	106 (85.3)	0.212
INR	1.66 (0.93)	1.63 (0.93)	1.78 (0.92)	0.196
PT	18.0 (10.5)	17.7 (10.9)	19.0 (8.41)	0.271
APTT	42.2 (27.9)	40.7 (25.9)	48.8 (34.5)	0.058
ALT	61.9 (135)	54.7 (103)	92.3 (224)	0.159
AST	96.8 (218)	87.9 (199)	134 (284)	0.184
TB	1.84 (4.39)	1.66 (3.60)	2.59 (6.74)	0.252
CRE	1.76 (1.41)	1.66 (1.38)	2.15 (1.44)	0.010
URE	37.4 (27.6)	34.2 (26.3)	51.0 (29.1)	<0.001
LDH	307 (185)	292 (158)	371 (263)	0.014
Sa:				1.000
No	131 (33.7%)	106 (33.8%)	25 (33.3%)	

	[ALL]	Survivor	No-survivor	P-value
	N=389	*N=314*	*N=75*	
Yes	258 (66.3%)	208 (66.2%)	50 (66.7%)	
Vp:				0.005
No	146 (37.5%)	129 (41.1%)	17 (22.7%)	
Yes	243 (62.5%)	185 (58.9%)	58 (77.3%)	
Gc:				0.378
No	273 (70.2%)	224 (71.3%)	49 (65.3%)	
Yes	116 (29.8%)	90 (28.7%)	26 (34.7%)	
ABX: Yes	389 (100%)	314 (100%)	75 (100%)	.
Ventilation:				0.393
No	53 (13.6%)	40 (12.7%)	13 (17.3%)	
Yes	336 (86.4%)	274 (87.3%)	62 (82.7%)	
CRRT:				0.056
No	356 (91.5%)	292 (93.0%)	64 (85.3%)	
Yes	33 (8.48%)	22 (7.01%)	11 (14.7%)	

Table S4: Coherent linear screening (VIF) in each queue

ICU-28Dead		Hosp_28Dead		external validation	
**Variable Names**	**VIF**	**Variable Names**	**VIF**	**Variable Names**	**VIF**
WBC	1.11	Age	1.91	Age	1.79
APTT	1.12	Hypertension	1.18	AKI	1.25
HLD	1.12	AKI	1.21	SOFA	2.45
Gc	1.13	HLD	1.10	APSIII	2.82
AKI	1.18	SOFA	2.55	SAPSII	4.17
K	1.20	APSIII	3.84	charlson	1.45
po2	1.23	SAPSII	4.12	APACHEII	2.70
CRRT	1.24	OASIS	2.34	RDW	1.31
ALB	1.29	charlson	1.60	ALB	1.38
Sa	1.31	APACHEII	2.84	APTT	1.22
HCT	1.32	NBPS	1.73	ALT	1.31
Hypertension	1.32	NBPD	1.67	TB	1.37

Vp	1.35	RDW	1.34	URE	1.57
ph	1.49	WBC	1.06	Vp	1.27
Lac	1.53	ALB	1.22	CRRT	1.29
RDW	1.54	AG	1.71		
CKD	1.67	K	1.19		
TCO2	1.68	TCO2	1.57		
NBPD	1.76	Lac	1.58		
NBPS	1.77	ph	1.41		
TB	1.77	po2	1.17		
AG	1.86	INR	43.88		
charlson	1.93	PT	43.52		
Age	1.98	APTT	1.24		
CRE	2.02	TB	1.60		
OASIS	2.25	URE	1.67		
SOFA	2.64	LDH	1.26		
APACHEII	3.07	Sa	1.40		
APSIII	3.80	Vp	1.37		
SAPSII	4.15	CRRT	1.17		
RBC	8.87				
HB	21.95				
PT	40.74				
INR	41.20				

TableS5: Statistical table of missing values for each variable

Variable Names	level	Overall	Missing
N		2593	
Age		73 (21-104)	0
Weight		77.9 (27.3-575)	4.1
height		170 (137-203)	47
SOFA		6 (2-21)	0
APSIII		55 (13-148)	0
SAPSII		43 (6-102)	0
OASIS		35 (12-64)	0
charlson		6 (0-18)	0
APACHEII		21 (3-52)	0
HR		90 (0-191)	0
NBPS		118 (41-230)	1
NBPD		65 (11-190)	1
RR		19 (0-63)	0.1
Spo2		98 (39-100)	0
temperaturef		98.2 (0-103.4)	0.5
absolute_lymphocyte_count		1.13 (0-222.85)	55.5
HCT		30.7 (10.6-57.6)	0.2

HB	9.9 (3.1-18.8)	0
PLT	189 (11-1379)	0
RDW	15.6 (11.7-29.3)	0.1
RBC	3.33 (0.9-6.47)	0
WBC	11.8 (0.1-506.6)	0.2
absolute_neutrophil_count	8.73 (0-44.24)	55.5
HbA1c	5.7 (4-14)	61.7
ALB	2.9 (1-5.5)	9.3
AG	15 (1-49)	0
Ca	8.3 (2-13.5)	0.4
Cl	104 (61-136)	0.1
GLU	133 (23-1027)	0
K	3.95 (1.6-9.3)	0.1
Na	4.1 (1.6-9.8)	86.8
TCO2	139 (98-176)	0.1
Lac	24 (4-61)	13.8
free_calcium	1.11 (0.71-1.66)	38.8
Lac	1.8 (0.3-20.6)	0
Pco2	40 (10-121)	3.9
ph	7.37 (6.84-7.66)	20.5
po2	83 (14-681)	7.9
ddimer	3312 (364-21240)	97
FIB	348 (29-1306)	73
INR	1.4 (0.9-15)	2.9
PT	15 (10-150)	2.8
APTT	32.5 (18.2-150)	3.3
thrombin	18.2 (12.7-150)	98.6
HDL	45 (10-117)	65.9
LDL	85.5 (9-321)	66.5
TC	156.5 (47-388)	65.4
TG	117 (13-2786)	54.2
ALT	24 (1-12940)	4.7
AST	38 (0-10760)	3.7
DBIL	1.15 (0.1-18.2)	89
IBIL	0.7 (0.1-15.3)	88.8
TB	0.6 (0.1-69.6)	3.7
CRE	1.3 (0.1-11.7)	0
URE	28 (1-235)	0.1
UC	4.6 (0.8-14.4)	92.7
CK	171.5 (9-72670)	41.2
CK-MB	4 (1-411)	52.3
LDH	264 (60-11995)	0
NT-PRO-BNP	2464 (25-63198)	90.5
troponin_t	0.08 (0-8.16)	73.6

lymphocytes		10.9 (0-100)	34.4
Mg		6.8 (0-46)	34.4
neutrophils		79 (0-96)	34.4
absolute_monocyte_count		0.81 (0-13.65)	55.5
hosp_day		13.09 (0.28-248.45)	0
icu_day		3.96 (0.03-86.85)	0
gender (%)	F	1476 (56.92)	0
gender (%)	M	1117 (43.08)	
Hypertension (%)	0	1689 (65.14)	0
Hypertension (%)	1	904 (34.86)	
Race (%)	Other races	880 (33.94)	0
Race (%)	WHITE	1713 (66.06)	
AKI (%)	0	981 (37.83)	0
AKI (%)	1	1612 (62.17)	
CKD (%)	0	1817 (70.07)	0
CKD (%)	1	776 (29.93)	
Diabetes (%)	0	1620 (62.48)	0
Diabetes (%)	1	973 (37.52)	
HLD (%)	0	1659 (63.98)	0
HLD (%)	1	934 (36.02)	
IHD (%)	0	1580 (60.93)	0
IHD (%)	1	1013 (39.07)	
COPD (%)	0	2139 (82.49)	0
COPD (%)	1	454 (17.51)	
Sa (%)	0	874 (33.71)	0
Sa (%)	1	1719 (66.29)	
Vp (%)	0	954 (36.79)	0
Vp (%)	1	1639 (63.21)	
Gc (%)	0	1746 (67.34)	0
Gc (%)	1	847 (32.66)	
ABX (%)	0	1 (0.04)	0
ABX (%)	1	2592 (99.96)	
Ventilation (%)	0	349 (13.46)	0
Ventilation (%)	1	2244 (86.54)	
CRRT (%)	0	2374 (91.55)	0
CRRT (%)	1	219 (8.45)	
death_hosp_28days (%)	0	2177 (83.96)	0
death_hosp_28days (%)	1	416 (16.04)	
death_icu_28days (%)	0	2148 (82.84)	0
death_icu_28days (%)	1	445 (17.16)	
